# The Influences of Impulsivity and Education Levels on Severity of Alcohol Dependence

**DOI:** 10.3389/fpsyt.2020.00737

**Published:** 2020-08-05

**Authors:** Ziqi Liu, Ruyan Luo, Rao Fu, Chenxin Yuan, Xueming Xu, Dongsheng Zhou, Min Zhao, Ti-Fei Yuan, Jiang Du

**Affiliations:** ^1^ Shanghai Key Laboratory of Psychotic Disorders, Shanghai Mental Health Center, Shanghai Jiaotong University School of Medicine, Shanghai, China; ^2^ Department of Substance Abuse and Addiction, Shanghai Mental Health Center, Shanghai Jiaotong University School of Medicine, Shanghai, China; ^3^ Department of Addiction, Taizhou Second People’s Hospital, Taizhou, China; ^4^ Department of Psychiatry, Ningbo Kangning Hospital, Ningbo, China; ^5^ Co-innovation Center of Neuroregeneration, Nantong University, Nantong, China

**Keywords:** alcohol use disorder, impulsivity, education, dependence, association

## Abstract

**Background:**

Impulsivity contributes to the severity of alcohol use disorder. The association is affected by expectation towards alcohol use, emotional regulation and self-control. Here we investigated the influences of self-reported impulsivity and levels of education on severity of alcohol dependence.

**Method:**

We retrospectively analyzed the basic demographic information, alcohol consumption state, education years, depression and anxiety state, Alcohol Use Disorder Identification Test (AUDIT) and Barrett Impulsivity Scales (BIS) from a group of 66 AUD patients.

**Result:**

Impulsivity significantly predicted alcohol dependence severity (*R* ^2^ = 0.069, *F* = 4.724, *p* = 0.034). In addition, education years served as a moderator in the relationship between impulsivity and alcohol dependence severity (*ΔR^2^* = 0.059, *F* = 4.414, *p* = 0.040).

**Conclusion:**

Self-reported impulsivity affects the severity of alcohol dependence, which might be different in patients with different education levels.

## Introduction

Alcohol use disorder (AUD) causes high social and economic burdens (1, 2). Impulsivity serves as vulnerable endo-phenotype for substance abuse, including AUD (3). In addition, personality traits such as neuroticism/negative emotionality and extraversion/sociability are linked to problematic alcohol use (4).

The interaction of high impulsivity trait with other risk-factors co-contributes to the severity of AUD (5). For instance, impulsivity mediates alcohol intake selectively in students with expectation of having drink with positive experiences (6). In addition, the emotion regulation ability significantly weakens the association between non-planning/attentional impulsivity and alcohol use disorder (7). Education alters knowledge, skill, emotional regulation ability, and changes the attitude towards substance abuse. One previous study reported that people with lower education years have higher risks of non-problematic heavy drinking and problem drinking (8).

The present study investigated the impact of self-reported impulsivity on severity of AUD patients, and examined if education levels might affect the association. We retrospectively analyzed 66 AUD patients from two hospitals for their demographic information, mood states, Alcohol Use Disorder Test (AUDIT) and Barrett Impulsivity Scale (BIS) scores.

## Method

### Participants

A total of 66 participants from two clinical sites (43 from Shanghai Mental Health Center, 23 from Taizhou Second People’s Hospital) between Jul 2018 and Aug 2019 were included for analyses. Inclusion criteria: (1) age above 18 years old; (2) diagnosed as alcohol dependence according to the Diagnostic and Statistical Manual of Mental Disorders, 4th edition (DSM-IV); (3) could complete the questionnaire under the help of professional doctor and postgraduate students of psychology or independently; (4) no psychoactive substance have been used before; (5) not co-morbid with other psychiatric disorders nor neurological disorders.

### Clinical Measurements

Participants’ demographic information, abstinence days, smoking (FTND), craving score, main alcohol beverage type, alcohol by volume (ABV) of their main alcohol beverage, the amount of alcohol consumption per day, Alcohol Use Disorder Test (AUDIT), Barrett Impulsivity Scale (BIS) were collected.

Fagerström Test for Nicotine Dependence (FTND) (Chinese version) contains six questions, scores ranging from 0 to 10. High score means high severity of nicotine dependent (9).

Craving score was assessed by the Visual Analogue Scale (10, 11). A line segmented into ten equal parts by 11 points (0–10) was presented to the participants. The leftmost point is “0” score, means “do not want to drink at all”, while the rightmost point is “10” score, means “extremely want to drink”. Participants were asked to mark one of those points on the line to represent their drinking desire intense.

Depression and anxiety state were measured by Beck Depression Inventory (BDI), Beck Anxiety Inventory (BAI) and Hamilton Rating Scale for Depression (HAMD), Hamilton Rating Scale for Anxiety (HAMA) (12–15). BDI and BAI are 21-items, four-point Likert scale ranging from 0 to 3, the total score is 63. HAMD and HAMA respectively contains 17 and 14 items. All items in HAMA and 9 items in HAMD are a five-point Likert scale ranging from 0 to 4, 8 items in HAMD are three-point Likert scale ranging from 0 to 2. The total score of HAMD and HAMA are 52 and 56. High self-report scale score indicates more server anxiety and depression.

The Chinese version of Alcohol use disorder Identification Test (AUDIT) was used (16), which could estimate the past-year alcohol dependence (17). It contains 10 items, the total score ranges from 0 to 50 (18). Scale over 8 score suggest for serious alcohol dependence.

Barrett Impulsivity Scale (BIS) contains three dimensions: non-plan impulsivity, motor impulsivity and attention impulsivity (19). As previously suggested, we report the final total BIS score as calculated by two steps. (1) each subscale score is equal to “[(sum of scores of each item − 10) ÷ 40] × 100”. (2) the total score is equal to “the sum of scores of three subscales/3”.

### Statistics Analyses

The data is processed with SPSS 21.The scores of AUDIT, BIS and each participants’ education years were centralized to reduce the multicollinearity (20). Hierarchical linear regressions were conducted to test the moderating effect of education years by two steps. First, the centralized scores of BIS and education years were taken as the predictor variables, and AUDIT score was used as the outcome variable. Second, we added the multiplication of the centralized score of BIS and education years. Moderating effect would be existed if the coefficient of the multiplication item is significant. The significance level alpha was set to 0.05 (two-tailed).

**Figure 1:**
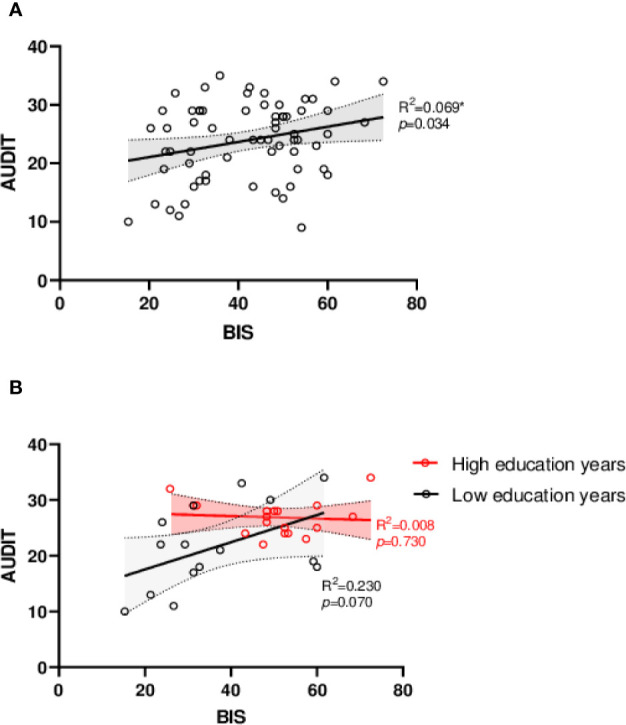
Linear regression diagram. **(A)** Linear regression analysis of BIS and AUDIT. **(B)** Linear regression analysis of BIS and AUDIT between different education attainments. Low education years: 0–6 years of education, primary school; High education years: above 13 years of education, college or university. AUDIT, Alcohol use disorder Identification Test; BIS, Barrett Impulsivity Scale. *p < 0.05, **p < 0.01.

We further conducted linear correlation analysis and made linear regression diagram. **Figure 1A** presents the correlation between BIS and AUDIT among all patients while **Figure 1B** presents with two groups of patients: (1) Low education years, patients had graduated or had not graduated from primary school, 0–6 years of education; (2) High education years, patients graduated from high school and had been admitted by college, above 13 years of education.

## Results

The demographic and clinical characteristics of the patients are presented in **Table 1**. 89.4% participants were male and the average age is 48.96 ± 12.45. They accepted average 10.09 ± 4.18 years of education. Patients self-reported they consumed average 773.86 ± 986.59 ml alcoholic beverage per day before hospitalized. Nineteen patients mainly drank 12.32 ± 3.62% beer or wine, 47 patients mainly drank 44.83 ± 7.10% liquor.

**Table 1:** Demographic and Clinical Characteristics of the Patients.

		Total n = 66	
		Mean	Stand deviation
Gender		Male: Female 59: 6	
Age		48.96	12.45
Education years		10.09	4.18
Abstinence days		89	184.03
ABV(%vol)	Wine or beer (n = 19)	12.32	3.62
	Liquor (n = 47)	44.83	7.10
ml/day		773.86	986.59
Days of withdrawal		89.09	184.10
Hight (centimeter)		170.12	6.19
Weight (kilogram)		65.29	12.00
FTND		4	2.95
Depression	BDI (n = 23)	10.22	9.21
	HAMD (n = 43)	5.81	5.00
Anxiety	BAI (n = 23)	25.04	4.57
	HAMA (n = 43)	5.53	5.14
AUDIT		23.92	6.56
BIS		41.96	13.24
Craving		5.09	2.81

ABV, Alcohol by volume; AUDIT, Alcohol use disorder Identification Test; BIS, Barrett Impulsivity Scale.

**Figure 2:**
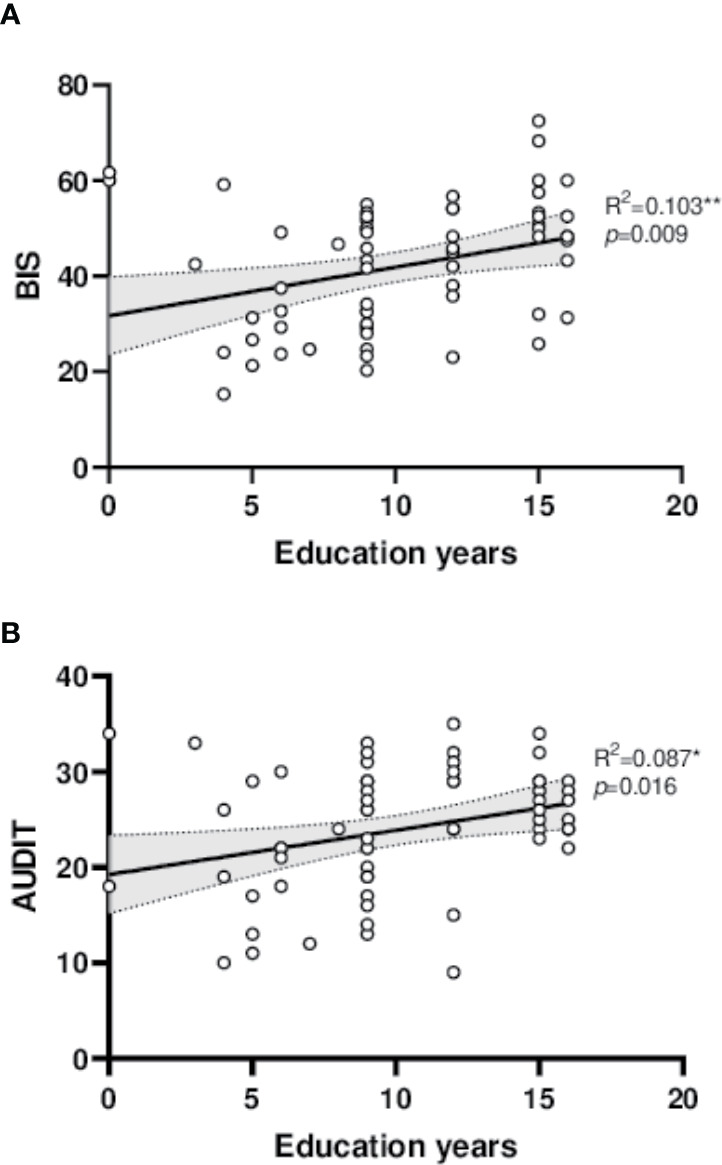
Linear regression diagram. **(A)** Linear correlation between education years and BIS. **(B)** Linear correlation between education years and AUDIT.AUDIT, Alcohol use disorder Identification Test; BIS, Barrett Impulsivity Scale. *p < 0.05, **p < 0.01.

Impulsivity and alcohol dependence severity presented significant correlation (*R^2^* = 0.069, *F* = 4.724, *p* = 0.034), higher impulsive trait related to more serious alcohol dependence (**Figure 1A**). The BIS-AUDIT slopes of low and high education years are different from each other (**Figure 1B**). Interestingly, increased education years also significantly related to higher impulsivity (*R^2^* = 0.103, *F* = 7.360, *p* = 0.009) and more severe alcohol dependence (*R^2^* = 0.087, *F* = 6.113, *p* = 0.016, **Figure 2**).

**Table 2** presents that education years is a significant moderator that could influence the relationship of BIS and AUDIT (β = −0.028, SE = 0.013, *p* = 0.040). For patients with low education years, high impulsivity results in more serious alcohol dependence; while in patients with high education years, the slope appeared nearly flat, implying a reduced relationship between impulsivity and alcohol dependence severity.

**Table 2:** Education years as moderator.

	Outcome variable	Predictor variable	Non standardized coefficient		Δ*R* ^2^	*F*	Sig.
			β	SE			
Step1	AUDIT	BIS	0.092	0.062	0.118	4.231	0.140
		Education years	0.369	0.196			0.064
Step2	AUDIT	BIS	0.065	0.062	0.059	4.414	0.293
		Education years	0.555	0.210			0.010**
		BIS*Education years	-0.028	0.013			0.040*

AUDIT, Alcohol use disorder Identification Test; BIS, Barrett Impulsivity Scale. *p < 0.05, **p < 0.01.

## Discussion

The study reported the association between self-reported impulsivity and clinical severity of AUD patients, and that education level might interact with this association. The results are in a line with previous findings that impulsivity contributes to substance abuse, and co-contributes to clinical symptoms with other demographic variables and personality traits. The findings might rationalize the utility of impulsivity-targeting therapy in selective population for individualized treatments.

The effects of education on impulsivity and drug dependence have been reported. For instance, it is proposed that alcohol consumption increases with the level of education and higher income (16). Higher educational attainment is associated with increased odds of daily alcohol consumption and problem drinking (21). High-school graduation and college graduation were associated with increased odds of ever drinking (22). Higher educational attainment could improve internal personal resources, change individual from complete aspects, develop knowledge, skills, motivation, self-esteem, personal disposition, self-control and executive function, facilitate the development of prefrontal cortex (PFC). Educational attainment also determines individuals’ socioeconomic status which affects alcohol dependence (23), and influences alcohol abuse by moderating the effect of genetic and environment interactions (24). All these variables could contribute to the observation in present study. The mechanism of becoming alcohol abuse is different for people of different educational level. For the highly educated patients, alcohol is the last option to release their pressure or pain. Once they release themselves by taking alcohol, they more easily to become serious addiction. While for low educated patients, drinking is more likely a dangerous entertainment. The more impulsive the individual is, the more likely he would prefer drinking, a risky and harmful entertainment.

It should be noted that education level is inversely associated with delay discounting performance (25). BIS and academic grades are inversely related (26). It is possible that highly impulsive subjects tend to terminate education earlier, which might bias conclusions based on direct analyses of education years.

The study has some limitations. First, the self-reported impulsivity with BIS psychometric assessment is limited (27). Sensation seeking, positive and negative urgency also closely associate with problematic alcohol use (28). Future study with laboratory-based behavioral tasks might improve the precision of measurements. Secondly, many demographic variables (e.g. fetal alcohol exposure, Attention Deficit Hyperactivity Disorder (ADHD), family alcoholism history and sociocultural environment) might affect the enrollment of patients, and these subjects should be recruited in future studies. Besides impulsiveness, other factors, such as stress, insomnia, mood disorder, chronic pain and post‐traumatic stress disorder (PTSD) also contribute to alcohol dependence, further studies should involve these dangerous factors. Thirdly, the sample size is relatively small, so we should take cautious view of the conclusion and need to be revalidated in other larger population sample. Finally, we also expect to verify the result from neurobiology perspective.

In conclusion, self-reported impulsivity affects the severity of alcohol dependence, which might be different in patients with different education levels.

## Data Availability Statement

The datasets generated for this study are available on request to the corresponding authors.

## Ethics Statement

The studies involving human participants were reviewed and approved by Ethics Committee of Shanghai Mental Health Center. The patients/participants provided their written informed consent to participate in this study.

## Author Contributions

MZ, T-FY, and JD designed the study. ZL, RL, RF, CY, and DZ performed the study and analyzed the study together. All authors contributed to the article and approved the submitted version.

## Funding

We are grateful for all participants who completed the survey despite their heavy workload. This work was supported by the National Key R&D Program of China (2017YFC1310400), the National Nature Science Foundation (U1502228, 81771436, 81871045, 81822017, 31771215), the Shanghai Academic Research Leader Program (17XD1403300), Shanghai Municipal Health and Family Planning Commission (2017ZZ02021, 2018BR21), Shanghai Key Laboratory of Psychotic Disorders (13DZ2260500), Shanghai Municipal Science and Technology Major Project (2018SHZDZX05), Science and Technology Commission Shanghai Municipality (19411969200), Shanghai Municipal Education Commission - Gaofeng Clinical Medicine Grant Support (20181715).

## Conflict of Interest

The authors declare that the research was conducted in the absence of any commercial or financial relationships that could be construed as a potential conflict of interest.
